# Supplementary figures and images for: Preferences for achromatic horizontal, vertical, and square patterns in zebrafish (Danio rerio)
Source: PeerJ. 2017 Sep 4;5:e3748. doi: 10.7717/peerj.3748 (PMC5588788; doi:10.7717/peerj.3748)

# 10 mm Condition

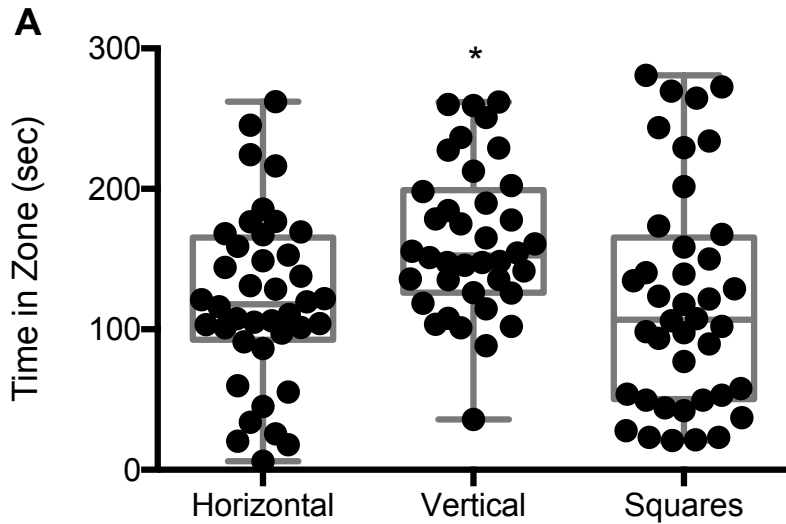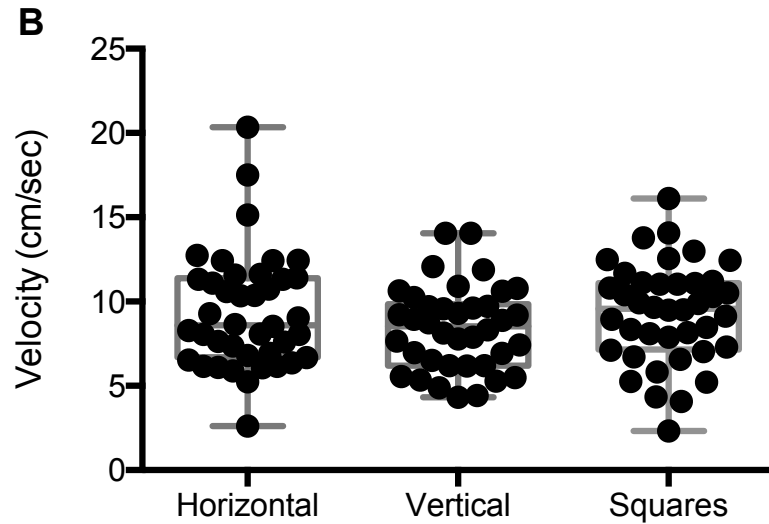

Supplement: Figure S1 — The horizontal line within the box indicates the median, the boundaries of the box indicate the 25th- and 75th -percentile, and the whiskers indicate the highest and lowest values of the results. All data points are represented as black dots superimposed on box. (A) Time spent in the zone with the indicated pattern. Fish spent significantly more time in the zone with the vertical stripes (p < 0.001). (B) Swim speed as measured by velocity of the fish within each of the pattern types. The swim speed of the fish did not differ between the three patterns. [file peerj-05-3748-s003.pdf]

# 5 mm Condition

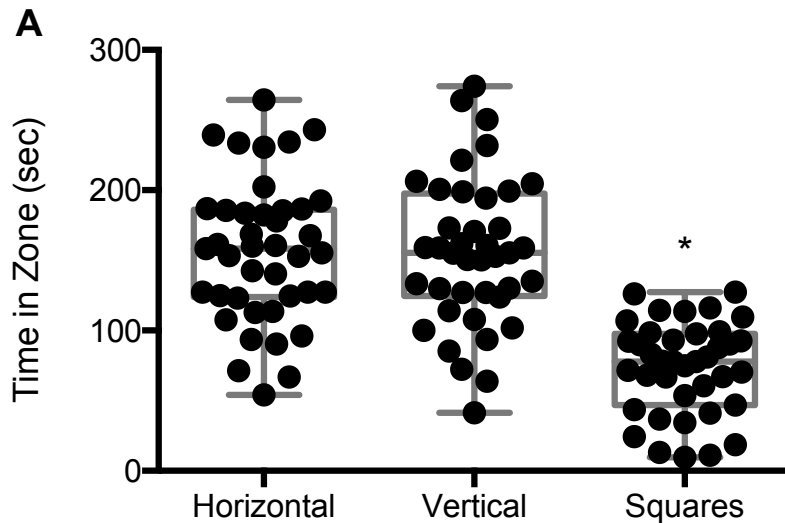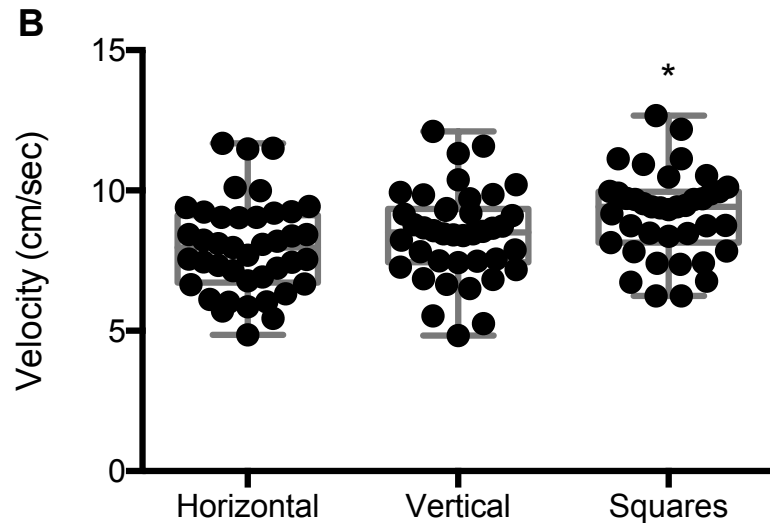

Supplement: Figure S2 — The horizontal line within the box indicates the median, the boundaries of the box indicate the 25th- and 75th -percentile, and the whiskers indicate the highest and lowest values of the results. All data points are represented as black dots superimposed on box. (A) Time spent in the zone with the indicated pattern. Fish spent significantly less time in the zone with the square pattern (p < 0.001). (B) Swim speed as measured by velocity of the fish within each of the pattern types. The fish swam significantly faster in the zone with the square pattern (p < 0.001). [file peerj-05-3748-s004.pdf]

# 1 mm Condition

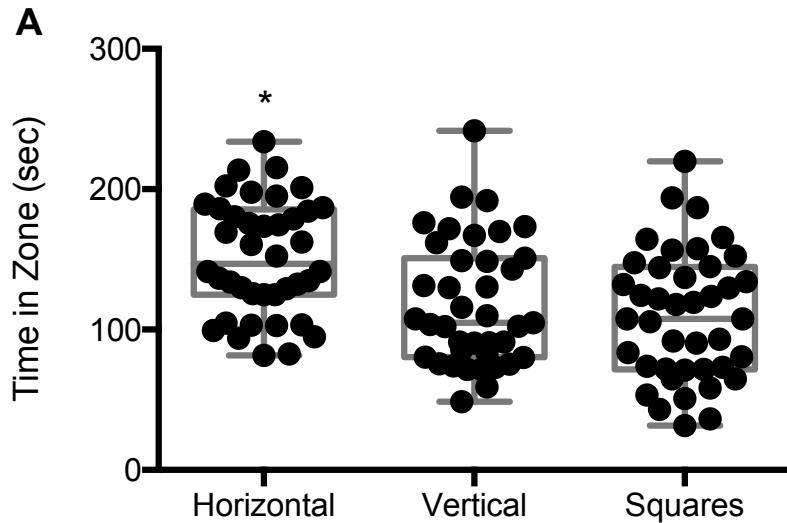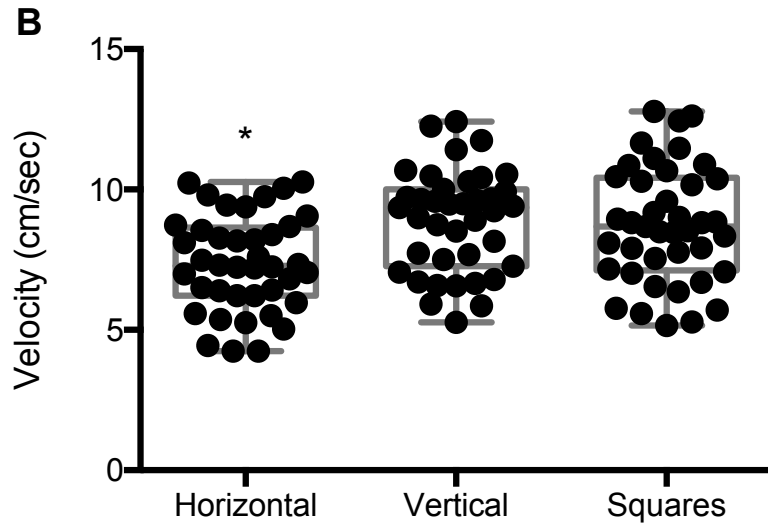

Supplement: Figure S3 — The horizontal line within the box indicates the median, the boundaries of the box indicate the 25th- and 75th -percentile, and the whiskers indicate the highest and lowest values of the results. All data points are represented as black dots superimposed on box. (A) Time spent in the zone with the indicated pattern. Fish spent significantly more time in the zone with the horizontal stripes (p < 0.001). (B) Swim speed as measured by velocity of the fish within each of the pattern types. The fish swam significantly slower in the zone with horizontal stripes (p < 0.05). [file peerj-05-3748-s005.pdf]
